# Understanding Aging Policies in China: A Bibliometric Analysis of Policy Documents, 1978–2019

**DOI:** 10.3390/ijerph17165956

**Published:** 2020-08-17

**Authors:** Yan Nan, Tieying Feng, Yuqun Hu, Xinzhu Qi

**Affiliations:** School of Public Policy and Administration, Xi’an Jiaotong University, Xi’an 710049, China; nanyanyan@yeah.net (Y.N.); huhu2628@stu.xjtu.edu.cn (Y.H.); qixinzhu@126.com (X.Q.)

**Keywords:** aging, policy evolution, bibliometric analysis

## Abstract

Aging poses a big challenge in all aspects of social governance in China. A coherent and focused aging policy response that spans multiple sectors of government has been undertaken to achieve the goal of “Healthy Aging”. From an historical perspective, this paper uses a bibliometric analysis method to probe into the evolution of Chinese aging policies from 1978 to 2019, and the roles of core government agencies in policy-making. We obtained 226 Chinese aging policies from the PKULaw Database and the websites of the government departments. Co-word analyses and network analyses were applied in mapping the topics of aging policies and collaboration among the agencies. Gephi software was used to visualize the most frequently used keywords and their network graphs. Findings are as follows. Firstly, the development of the aging policy system in China has undergone two phases, from focusing on basic security to emphasizing the rights and health of the elderly. Secondly, the network structure of aging policy-making departments presents a distinct edge-core layer. More and more government agencies have become involved in the formulation of aging policies. But collaboration among the agencies is insufficient. Thirdly, pilot promotion is the main tool for implementing aging policies.

## 1. Introduction

Aging around the world poses a global challenge in all aspects of social governance. Governments need to take steps to deal with the consequent demands for elderly services, long-term care services and related social assistance. Policy is the main tool for governments to build a sustainable governance structure [1]. Globally, a coherent and focused aging policy response that spans multiple sectors of government has been undertaken. Existing studies in the field mainly concern the introduction of aging policies in different regions or countries. Scholars have been primarily focused on residential aged care policies in Australia [2], the recent history of pension policies in the United Kingdom [3], aging and elderly care in the Arab region [4] and aging and health policies [5] and old-age adaptation policies in Europe [6]. Bloom et al. [7] and Feng [8] discussed the guiding principles of aging policies from the perspective of policy-making. Calvo et al. [9] used a comparative analysis to review and analyze aging policies in Argentina, Chile, Costa Rica and Mexico. Further, a qualitative case study method was employed in the above literature. Some scholars have increasingly turned their attention to the historical pattern of aging policy changes, trying to explain the policy characteristics and dynamics over time [10,11,12].

Bibliometrics have been applied to reveal development trends in certain areas of research [13]. Bibliometrics have also been widely used in policy studies, especially in analyzing policy changes [14,15,16]. The bibliometric analysis of policy documents involves different policy fields, such as the study of disaster management policies [17], science and technology policies [18], information policy [1] and so forth. Co-word analyses or social network analyses have also been applied in policy studies [19,20]. Therefore, bibliometric methods can also provide an insight into China’s aging policies. Although many papers related to the many aspects of aging policies have been published, policy dynamics have not been systematically investigated based on a bibliometric analysis. To fill this research gap, we performed a bibliometric analysis to probe into the evolution of China’s aging policies from 1978 to 2019 and the roles of core government agencies in policy-making.

The United Nations maintains that a country with more than ten percent of its population over 60, or seven percent of its population over 65, is an aging society [21]. China is aging at an unprecedented rate [22] and currently approximately 249.49 million people, or 17.9% of the total population, are 60 years or older [23]. The Chinese government needs to adjust their old policies or make new policies to deal with the severity and complexity of the aging society. This requires a historical understanding of current policies. Before the 1980s, the age structure of China’s population was still young. All sectors of society did not fully understand the process and consequences of population aging, and aging work was not included in the government agenda. Therefore, there was no systematic aging policy in China during this period [24]. After the third plenary session of the 11th CPC Central Committee in 1978, China reestablished the Ministry of Civil Affairs to resume its functions in charge of social assistance, social welfare and entitled groups. China’s social old-age security system returned to normal operation, and the problem of aging attracted the attention of central and local government [25]. Through a bibliometric analysis of documents on aging policies, this article analyzes the dynamic changes and trends in China’s aging policies since 1978. It aims to study China’s aging policy changes from an historical perspective in order to facilitate an understanding of the evolving process of the aging society management system in China and to explore future pathways of transformation. The paper addresses the following specific questions:

- How have China’s aging policies changed since 1978?
- What are the aging policy priorities in the different historical phases of the Chinese national agenda?
- What are the changes to the government departments involved in aging policy-making network and their interdepartmental relations?

## 2. Materials and Methods

The aging policy document data were derived from the PKULaw Database. The PKULaw Database is the largest and continuously updated Chinese policy full-text database that compiles public polices promulgated in mainland China since 1949. A full text retrieval was performed in the PKULaw Database on 2 January, 2020. Then we used the official government websites as supplementary databases for data validation, testing whether the selected policy samples covered all Chinese aging policies. Because Chinese aging policies emerge from a tightly bound government decision-making network, we searched the websites of all the Chinese central government departments, including the State Council, the Ministry of Civil Affairs, the Ministry of Finance and other departments (see Table 1). “Aging” (LAOLING) or “the elderly” (LAONIANREN) were used as the keywords when searching the databases. The search spanned from 1978 to 2019. A total of 489 central government documents related to aging were collected in this research.

To ensure the accuracy of the data, several criteria were adopted to filter the collected policies in this paper. The criteria were as follows: (1) three researchers worked together to determine whether the chosen policy was appropriate; (2) the duplicated policies were removed; (3) the policies whose keywords appear as phrases without any substantive information related to aging were excluded; (4) the policies should be in the form of a law, regulation, opinion, measure, notice or other document representing government policy, excluding industrial standard and supervision and reviews issued by social organizations; (5) the selected policies were national policies issued by the central government or its directly affiliated institutions; (6) the policies that had no guiding value and actual content or policies that were not closely related to aging and older people were removed. Based on the relevance of policy content and the suitability of policy types, we selected 226 final documents after cleaning the data. All the selected policy documents related to the governance of the aging society, and each document included the policy-making department, level of authority, regulatory category, and year and effectiveness status. The policy types included laws, opinions, notices, regulations, administrative orders, decisions, measures, rules, methods and outlines.

The bibliometric analysis method was used in this paper. First, we analyzed the temporal distribution and characteristics of aging policies in different phases. Second, a co-word analysis and cluster analysis were applied to discover the hotspots and the main directions of the aging policies. Co-word analyses aim to identify co-words and the co-absence of keywords [26] and to measure the strength of the relationships among the keywords. There are three main steps in this process: (1) extracting keywords from policy documents, (2) using Bibexcel to build a co-word matrix and (3) utilizing the social network analysis (SNA) to conduct a visualized atlas analysis. Gephi software was run to generate a visualization map of the keywords and the agencies to clarify their connections.

## 3. Results

### 3.1. Temporal Distributions

**Figure 1:**
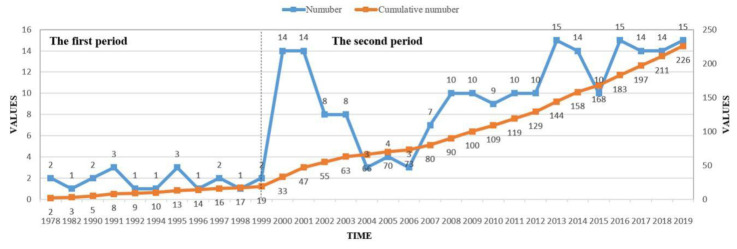
Phases and time distribution of China’s aging policies, 1978–2019.

Figure 1 illustrates the annual number of aging policies issued from 1978 to 2019, representing different growth trends in two phases. The dataset covers 226 documents over a 41-year period. From 1978 to 1999, the number of aging policies released each year remained steady, without significant changes. In 1999, the number of aging policies started to increase. From 2000 to 2019, approximately 10 policies on average were issued each year. As noted in the “Decision of the CPC Central Committee and the State Council on Strengthening work on Aging” and “the tenth Five-year Plan on Aging in China”, the governance of aging society entered a new stage in 2000. The “tenth Five-year Plan on Aging in China” also states that in the 21st century the Chinese central and local governments should issue a series of policies to solve the aging problems to meet the growing material and cultural needs of the elderly and to promote social equity and stability. Based on the changing trend of policy quantity and the expression of Chinese government official documents, the time-of-issue distribution of the policies can be divided into two sub-periods: Phase I lasted from 1978 to 1999; Phase II extended from 2000 to 2019. The following policy bibliometric analysis is based on these two historical phases.

### 3.2. Emphasis and Changes in Policy Content

**Figure 2:**
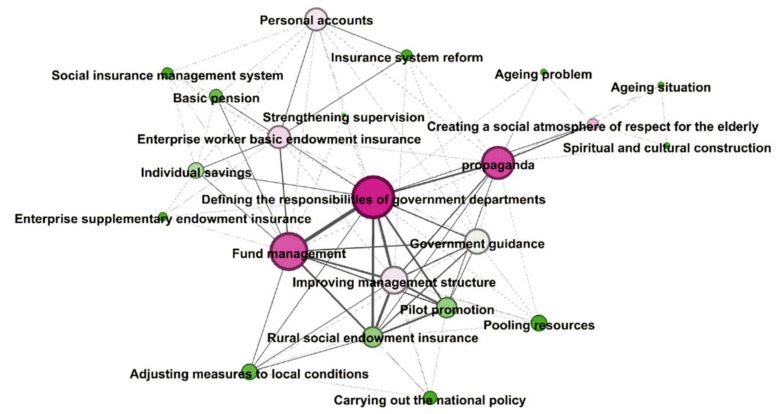
Keyword network of aging policies during Phase 1 (1978–1999) in China.

**Figure 3:**
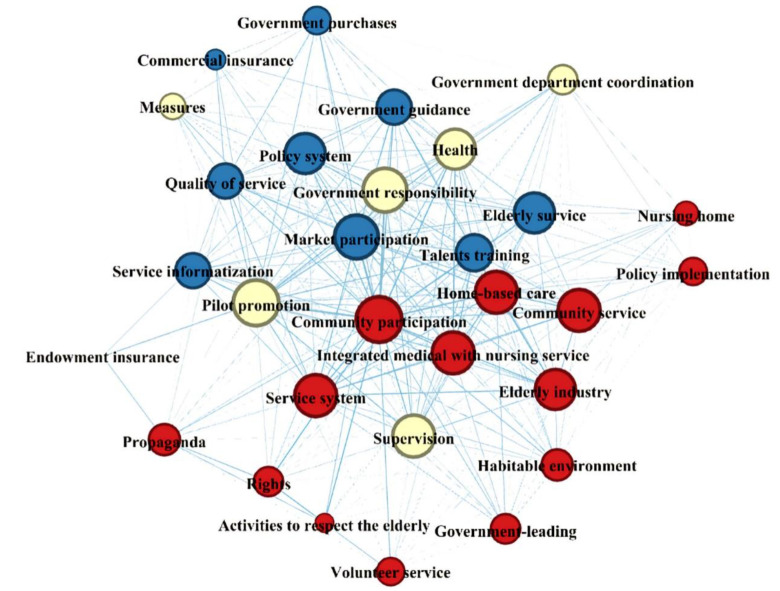
Keyword network of aging policies during Phase 2 (2000–2019) in China.

The policy topics changed across the two stages. The temporal features of the aging policy content are analyzed in this section. We identified 2 to 8 keywords for each policy document, screened out the high-frequency (In the top 20%) keywords during each stage, and then constructed a keyword co-occurrence matrix. This co-occurrence matrix should reflect the main interest areas and concerns of the policies. Gephi software was used to visualize the most frequently identified keywords and their network graph during the two stages. Figure 2 and Figure 3 present the two co-word networks for the two sub-periods. In the networks, the size of the nodes represents the frequency of the keywords, while the thickness of the lines indicate the strength of the co-occurrence of the keywords. All keywords of the same cluster appear as nodes with the same color.

#### 3.2.1. Phase 1: exploratory stage (1978–1999)

In the period of 1978–1999, China’s aging policies had three broad theme areas (Figure 2). The first two areas were focused on “enterprise worker endowment insurance” and “rural social endowment insurance”. The last area focuses on “insurance fund management”. “Enterprise worker endowment insurance” includes “enterprise worker basic endowment insurance” and “enterprise worker complement endowment insurance”. This characteristic indicates that endowment insurance is a key theme for Chinese aging policy related to the benefits of current and future retirees. China has legislated a mixed social security pension system during the exploratory stage of its aging policy, stipulating the public and private sources of pension funds to meet the high medical, care and maintenance costs of the elderly in city. “Decision on the reform of enterprise worker basic endowment system”, issued by the State Council in 1991, established that the government, enterprises and individuals jointly bear the financing of the basic old-age insurance. “Decision on establishing a unified system of enterprise worker basic endowment insurance”, issued by the State Council in 1997, laid out the “three-part pension system”, including the basic pension plan, the defined-contribution funded system and voluntary pensions. Enterprises contribute 22% of total employee wages to the basic pension system. Employees who have contributed to the pension system for 15 years can receive a basic pension equivalent to 20 percent of the average wage in the region after retirement. In “the defined-contribution funded system’’ section, municipal and provincial authorities establish an individual account for each employee. Employees and their enterprises contribute 11% of workers’ wages to the account. The voluntary pensions includes enterprise pension plans, individual retirement plans and other pension plans. For the elderly in rural areas, the expenditure of endowment expenses still come mainly from the families. However, some policies that explain the additional support for the rural elderly have been issued during the exploratory stage. “Basic plan for rural social endowment insurance (trial)”, issued by the Ministry of Civil Affairs in 1992, accelerated the construction of the rural social endowment insurance system. The plan states that all individual contributions and collective subsidies are put into individual pension accounts. Those farmers who participate in the insurance can receive a pension after the age of 60, according to the accumulation of funds in individual accounts and the corresponding calculation formula. But the pension system is mainly contributed by individuals, and the degree of pension security in rural areas is relatively low.

This characteristic is also linked to China’s economic system reform and institutional restructuring. In 1978, under the background of Reform and Opening Up, China began to explore the socialist market economic system. The insurance system, which was supported by the state in the era of planned economy, was reformed accordingly. Enterprise workers and farmers were the two major groups involved in endowment insurance. In 1994, the rural social insurance department belonging to the Ministry of Civil Affairs was established to administer rural social insurance. As a result, several policies on enterprise worker endowment insurance and rural endowment insurance emerged. “Decision on establishing a unified system of enterprise worker basic endowment insurance”, issued by the State Council in 1997, and “Notice on further strengthening rural social endowment insurance work”, issued by the Ministry of Civil Affairs in 1991, were two important policies in this phase. In addition, “fund management” is a key aging policy topic during this period for the following reasons. The endowment insurance fund is the fund reserve to pay workers and farmers when they are old. Insurance fund safety concerns whether old people can receive endowment insurance on time. For example, “Notice on strengthening the collection and management of the endowment insurance fund”, which was issued in 1990, emphasizes that endowment insurance fund management is crucial to safeguard retired workers’ lives and to maintain social stability. The focus is also on topics at the edge of the network such as the “aging situation”, “aging problem” and “insurance system reform”. This shows that aging problems emerged in China during this period and caught the attention of government departments.

The map presented in Figure 2 also shows the keyword clusters of the aging policies (1978–1999). The red cluster (center) includes keywords related to policy tools. Major words are as follows: defining the responsibilities of government departments, fund management and propaganda. It shows that the aging policies of Phase I (1978–1999) emphasize ensuring the safety of the pension fund under the guidance of government departments. Propaganda is also often used. The light green groups are larger but looser groups. Major words include the following: pilot promotion, rural social endowment insurance, pooling resources, adjusting measures to local conditions, carrying out the national policy and so forth. These words illustrate the importance of policy experiments related to rural social endowment insurance. On the basis of adjusting measures to local conditions, Chinese government departments hoped to carry out reform of the rural social endowment insurance system through strengthening policy-making and through investing more resources. Finally, the pink cluster illustrates the policy measures related to the pension insurance of enterprise employees. Major words and phrases are enterprise worker basic endowment insurance, personal accounts and improving management structure.

#### 3.2.2. Phase 2: Development Stage (2000–2019)

From 2000–2019 (Figure 3), the main focus was on topics such as “community participation”, “market participation”, “elderly services” and “elderly health”. Along with the growth of policies during this phase, the number of edges also increased. This contributes to the growth in the size of the keyword network. The aging policy system entered a period of initial development.

When comparing the keyword network diagrams of stage 1 and stage 2, we find several significant changes. First, the word “service” including “elderly service”, “service information”, “service system” and “quality of service” appears frequently in the period from 2000 to 2019, while “endowment insurance” only appears once at the edge of the network. This finding suggests that the policy focus has shifted from the pension system to the elderly service system. Second, “government responsibility”, “government guidance”, “government purchase”, “community participation” and “market participation” appear simultaneously as policy instruments, meaning that multiple subjects jointly participate in providing elderly services. Meanwhile, marketization programs rise as a recognizable force in aging policies. Last, “home-based care”, “integrated medical with nursing service” and “community service” appear in phase 2 as emerging trends in aging policies. These changes reflect China’s active response to an aging society. China became an aging society in 2000. Precise elderly service policies and more efficient policy measures were urgently needed. The changes in keywords based on the maps of the two stages exhibit that the term “elderly service” appears with time. The two opinions, accelerating the Development of the Elderly Service Industry and Promoting the Development of Elderly Care Services, were issued in 2013 and 2019, respectively. The former mentions that the Chinese government should develop a home-based elderly care network, flourish the consumption market for elderly services and promote the combination of medical and nursing services. The latter stipulates a deeper reform of decentralization, supervision and service, and expanding investment and financing channels for elderly services [27]. “Elderly service system” is also a hot keyword. Since the State Council proposed the construction of social elderly services in 2011, the Chinese government has endeavored to build an elderly service system. “Implementation Opinions on Supporting Social Elderly Care Service System Construction with Development Finance”, issued in 2015, emphasizes the role of development finance in guiding the construction of a social elderly service system. “Healthy China strategy” in aging policies was initially put forward at the 19th National Congress of the CPC in 2017, and “elderly health” immediately became a popular topic. Afterwards, a series of healthy aging programs were launched in order to improve the quality of life of the elderly in China. In addition, the keywords “right” and “habitable environment” reveal that protecting elderly people’s rights and the construction of a suitable environment were also the focuses of aging policies during this stage.

In the keyword network visualization map of the aging policies during Phase 2 (2000–2019) (Figure 3), the color of an item was determined by the cluster to which the item belonged. Thus, there are three clusters in the map: cluster 1 (red points), cluster 2 (yellow points) and cluster 3 (blue points). To better grasp the content of each cluster, we traced the source of each keyword and read the original policy texts to describe and analyze the meaning of the cluster. The main idea of cluster one is elderly service system construction. Major words are “service system”, “integrated medical with nursing service”, “community service”, “home-based care”, “government-leading” and “policy implementation”. The construction of an elderly service system is a complicated project. Family, community and nursing rooms are regarded as the main providers of elderly services. Additionally, the core subject is the government. Meanwhile, the combination of medical with nursing care is necessary to improve the health conditions of the elderly. Healthy aging is the emphasis of cluster two. Major words are elderly health, government responsibility, government department coordination, supervision, pilot promotion and measures. China’s healthy aging project is still in its infancy. The government had to clarify the responsibilities of the relevant departments and encourage them to coordinate with each other. Pilot promotion is the main means of the elderly health project. Cluster three primarily includes measures to improve elderly service quality. Major words are “elderly service”, “quality of service”, “government purchases”, “policy system” and “market participation”. These policies call for a focus on improving the quality of elderly services through the simultaneous participation of the government and the market as well as through using information technology. A perfect policy system is also important for achieving this goal. Therefore, specific means to improve the quality of elderly services is reflected in cluster three.

### 3.3. Network of Core Aging Policy-Making Departments

The number of and the connections between aging policy-making departments can be discussed in general through determining the number of policies issued separately or jointly. Forty three agencies have been involved in issuing aging policies since 1978 (Table 1).

#### 3.3.1. Phase 1: Exploratory Stage (1978–1999)

As shown in Table 2, only six departments were involved in the formulation of aging policies between 1978 and 1999. The Ministry of Civil Affairs issued the most documents, followed by the State Council. It is important to note that all departments are not linked to other members. This is because during this period, the issue of aging had just entered the policy agenda and had not yet aroused widespread concern. Civil affairs departments mostly carried out the basic work before China became an aging society in the form of notices and opinions, while other departments did not have specific arrangements.

#### 3.3.2. Phase 2: Development Stage (2000–2019)

**Figure 4:**
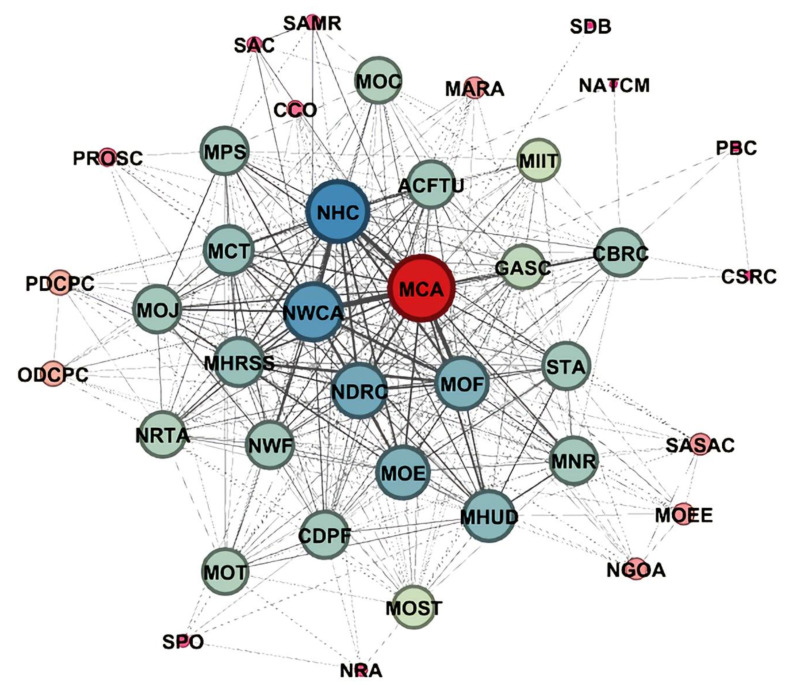
Network of aging policy-making departments during Phase 2 (2000–2019) in China.

Between 2000 and 2019, more and more agencies became involved (Figure 4) and they collaborated extensively. Figure 4 presents the visualization of the aging policy-making department network during the period. We imported the co-occurrence matrix of aging policymakers into the Gephi software and used the force atlas algorithm to map the network of policy-making departments. This network graph is undirected. For the network visualization, the individual policy-making departments are the nodes and the co-policymakers are displayed as linking lines or edges. The size of the nodes represents the number of cooperative policies issued by a single agency, and the thickness of the edge reflects the number of co-policymaker links between a given set of departments/nodes. The aging policy-making network has 39 nodes, 356 edges, a diameter of 2, an average path length of 1.53 and an average clustering coefficient of 0.885. The network is concentrated, which indicates that the cooperative relationship between various departments is stable and reliable.

The network structure presents a distinct edge-core layer. The Ministry of Civil Affairs takes the central position in the collaborative network, as it is the main department responsible for aging affairs. The National Working Commission on Aging, National Health Commission and Ministry of Finance are located very close to the network center, which indicates their frequent cooperation with other policy-making departments. Meanwhile, the Ministry of Civil Affairs, the National Working Committee on Aging and the National Health Commission are the most closely connected sub-network. This may be explained by the fact that aging policies changed and became more inclusive during this period. On the one hand, the National Health Commission is in charge of the rehabilitation, nursing care and spiritual comfort of the elderly. On the other hand, a variety of elderly service supply models, such as government-purchased services, subsidies and discounted interest, require the guidance and support of the Ministry of Finance. Departments such as the Ministry of Ecology and Environment, the Poverty Relief Office of the State Council and the National Railway Administration are located on the edge of the collaborative network because they are only responsible for some aspects of aging affairs. For example, the Poverty Alleviation Office of the State Council only focus on poverty alleviation for poor elderly people. In addition, the participation and cooperation of the Ministry of Housing and Urban-Rural Development, the Ministry of Industry and Information Technology, the Standardization Administration and the Ministry of Human Resources and Social Security provide land, capital, information, services, human resources and other resources for creating elderly services [28]. These departments are at the middle level of the network. It is interesting to see that the State Council does not appear in the aging policy-making department network, which indicates that it has not issued collaborative aging policies. The SNA analysis of the degree of centrality identified that the State Council scored 0, ranking it last (Table 3). This is determined by the nature of the State Council. The State Council, China’s highest administrative body, often issues independent, macroscopic and instructive policies. Other departments make overall arrangements for specific matters under its policy guidance.

## 4. Discussion

### 4.1. Underlying Pattern

The number of aging policies in China increased rapidly since 2000. This development of aging policies is consistent with the progress of China’s aging society, revealing the strong need for more guidance on aging and for improved preparation for future demographic trends. Since China became an aging society in 2000, the government has faced the dual pressures of insufficient labor supply [29] and an increased pension burden [30]. Building a systematic aging policy system is an important task for many Chinese government departments at this stage [31,32]. Indeed, many other countries are confronted with similar aging challenges, and elderly service have become an urgent issue in policy discussion on a global scale [33]. As is the case in China, in the context of aging, numerous countries recognize the need for aging policies, and possess formal policies and strategies to solve the aging problems in the coming decades [34,35,36,37]. The development of the elderly service system in China has undergone two phases. The main direction of aging policies has shifted from focusing on basic security in the phase of 1978–1999, to emphasizing high-quality elderly services in the phase of 2000–2019. China’s aging policies throughout the two phases have focused on achieving the following objectives: ensuring the basic livelihood of the elderly, increasing subjective well-being and realizing the rights of the elderly. Observed trends in aging policy emphasis are similar in other middle-income and low-income countries. For example, aging policies in Argentina and Mexico are characterized by a gradual shift from economic security towards ensuring social rights and quality of life in the 20^th^ century [9]. However, the policy trends vary in forming pathways due to differing contextual influences. The Chinese aging policy characteristics at each stage originate from political, socioeconomic and cultural contexts as well as from the diverse needs of the elderly. The policy theme of the second stage is innovative and is based on the continuation of the first stage. Each stage also experiences important institutional changes and core policy agency changes, owing to the ministry system reforms and other social security policy adjustments. China’s policymakers can learn useful lessons from previous patterns of aging policies by considering their shortcomings and improving on them.

### 4.2. Room for improvement

Although the aging policies in China reflect progression from a basic living needs perspective to a high quality services-based approach, our quantitative analysis of the policy documents indicates that there is still plenty of room for improvement in the future. Keywords such as health, home-based care for the elderly and community care for the elderly appeared in the second phase of the policy documents, reflecting new trends (the Community Aging in Place) in elderly care services. This trend is largely in response to the general preference of most older people for “aging in place”, which means receiving care at home or in the community rather than in institutions [38]. “Aging in place” can reduce the impact of disability among low-income older adults by addressing individual capacities and the home environment [39,40]. Efforts to ensure the implementation of an “aging in place” policy have emerged in some countries [41,42]. However, they are not enough to meet the diverse needs of the elderly. “Elderly mental health”, “elderly education” and “human capital development” have not become important policy keywords, which is not in line with global efforts to promote active aging [43,44]. Active aging is defined as “the process of optimizing opportunities for health, participation and security in order to enhance quality of life as people age” [45]. The active aging perspective calls for the development of policies for citizens of all ages without segregation and to provide opportunities to improve the well-being of older people [46].

In addition, each country has emphasized specific aging issues. In China, the feeling of loneliness experienced by elderly people is a serious social problem. In China, 24.78% of the elderly have varying degrees of loneliness, among which the elderly in rural areas, women, those living alone and the disabled have the highest degrees of loneliness [47]. The Chinese government needs to reflect the vision of reducing the loneliness among the elderly in aging policies. Low birthrate is also a serious problem in an aging society. Although the universal two-child policy has slowed down the decline of the total population and labor force to some extent in China, some predicted results also showed that a decreasing trend of the total population has not changed [48]. Thus, it is necessary to deal with the low birthrate phenomena in aging policies and to emphasize it more than before [49].

Last but not least, some government agencies on the fringes of the aging policy-making department network have little contact (Figure 4), reflecting the insufficient collaboration among the agencies involved. Strengthening interdepartmental collaboration is still needed [50]. Pluralistic cooperation should be extended to more organizations, including health care organizations, social service agencies and international research centers [51,52]. Policymakers, researchers and service providers should seek efficient programs, resources and devices to meet the elderly’s needs but also their wishes, expectations and rights [53].

### 4.3. Limitations

By using bibliometric methods, our study clearly shows and analyzes the evolution of China’s aging policies from 1978 to 2019 and the role of core government agencies in policy formulation. Nevertheless, this paper has limitations. Policy documents can only represent a simple network of policy-making partnerships. In fact, cooperation in policy-making among government departments is more complex. Government agencies need constant interaction, communication and learning. However, this relationship cannot be reflected in the network established through a quantitative analysis of policy documents.

## 5. Conclusions

This study probes into the evolution of Chinese aging policies from 1978 to 2019, and the roles of core government agencies in policy-making. Based on the results across two stages of China’s aging policy-making, we may draw several key conclusions about policy changes in the context of rapid aging.

In terms of the number of policies issued each year, aging policy has entered into a new stage since 2000. The development of the aging policy system in China has undergone two phases, an exploratory stage (1978–1999) and a development stage (2000–2019). Compared to the relatively small number of policies issued during the first period, there is a numerical increase in the second period. The growth in the number of aging policies indicates that aging problems have received increasing attention from the Chinese government, especially after China entered the aging society.

The aging policy priorities change over time according to the Chinese government’s strategies and plans, from focusing on basic security to emphasizing the rights and health of the elderly. With the improvement of living standards and the increase in progressive thinking, the needs of the elderly have risen from a basic level to a higher level. In response to these changes, policy priorities have been adjusted, which is reflected in the emergence of keywords such as “health” and “rights”. Moreover, the government plays a central role in the provision of elderly services, but elderly services have taken on the characteristic of marketization. “Market participation” is at the center of the network in the aging policy keyword network of Phase 2 (Figure 3). This policy trend on the one hand saves government pension expenditures, but on the other hand improves the quality of elderly care services by introducing market competition. Finally, pilot promotion is the main tool for implementing aging policies. The result of policy content analysis shows that “pilot promotion” is another focus of policy and appears in both phases of the content network. Policy pilots are policy experiments with Chinese characteristics, which can reduce the costs of policy implementation and the risk of policy failure.

Regarding the proportional distribution and interdepartmental relations of aging policy-making agencies, increasing numbers of government departments are becoming involved in formulating aging policies, and the cooperation network has been strengthened. Aging policies are related to all aspects of social life, including health care, housing and education. To meet the multi-level needs of the elderly, all relevant government agencies must formulate corresponding policies and they should form cooperative mechanisms to ensure policy coordination, which is embodied in the principle of joint policy-making.

Our study hereby intends to promote knowledge and understanding of the Chinese aging policies from 1978 to 2019 and the relationships between the aging policy-making agencies in China through a bibliometric analysis method. Our results can provide guidance for the improvement and optimization of Chinese aging policies, and have important policy implications for “healthy aging” and “active aging”. Lessons from the results of bibliometric analysis may be relevant for supporting the aging policy system in China as well as in other countries.

## Figures and Tables

**Table 1:** The Core Policy-making Agencies on Aging.

Policy-Making Agency	Acronym
State Council	SC
Ministry of Civil Affairs	MCA
Ministry of Agriculture and Rural Affairs	MARA
National Working Commission on Aging	NWCA
Ministry of Human Resources and Social Security	MHRSS
National Development and Reform Commission	NDRC
Ministry of Finance	MOF
Ministry of Industry and Information Technology	MIIT
State Taxation Administration	STA
Ministry of Education	MOE
Ministry of Public Security	MPS
Central Committee of the Communist Youth League	CCCYL
Standardization Administration of China	SAC
National Radio and Television Administration	NRTA
General Administration of Sport of China	GASC
National Government Offices Administration	NGOA
State Development Bank	SDB
State Administration for Market Regulation	SAMR
National Railway Administration	NRA
National Health Commission	NHC
State Post Office	SPO
National Administration of Traditional Chinese Medicine	NATCM
Poverty Relief Office of the State Council	PROSC
State-owned Assets Supervision and Administration Commission	SASAC
Ministry of Transport	MOT
Ministry of Science and Technology	MOST
National Women’s Federation	NWF
Ministry of Commerce	MOC
Ministry of Ecology and Environment	MOEE
Ministry of Justice	MOJ
Ministry of Culture and Tourism	MCT
Publicity Department of the Communist Party of China	PDCPC
Organization Department of the Communist Party of China	ODCPC
Ministry of Housing and Urban-Rural Development	MHUD
Ministry of Natural Resources	MNR
People’s Bank of China	PBC
China Disabled Persons’ Federation	CDPF
China Banking Regulatory Commission	CBRC
China Securities Regulatory Commission	CSRC
All-China Federation of Trade Unions	ACFTU
Central Civilization Office	CCO
National People’s Congress	NPC
Ministry of Information Industry(cancelled)	MII

**Table 2:** Ranking of Agencies Identified with High Frequency Among the Aging Policies During Phase 1 (1978–1999) in China.

Department	Number of Aging Policies Issued	Proportion
Ministry of Civil Affairs	8	42.1%
State Council	5	26.3%
National People’s Congress	3	15.8%
People’s Bank of China	2	10.2%
Ministry of Information Industry	1	5.2%

**Table 3:** Aging policy-making departments during Phase 2 (2000–2019) in China: Social Network Analysis centrality analysis.

Department		1	2	3
Ranking		Degree	Nrm Degree	Share
21	MCA	110.000	21.696	0.086
24	NWCA	99.000	19.527	0.078
12	NHC	88.000	17.357	0.069
1	MOF	77.000	15.187	0.061
5	NDRC	69.000	13.609	0.054
25	MHRSS	61.000	12.032	0.048
19	MOE	59.000	11.637	0.046
30	MCT	52.000	10.256	0.041
39	MHUD	51.000	10.059	0.040
28	MOJ	47.000	9.270	0.037
23	NWF	46.000	9.073	0.036
37	ACFTU	44.000	8.679	0.035
10	STA	40.000	7.890	0.031
3	MPS	38.000	7.495	0.030
40	MNR	38.000	7.495	0.030
33	CDPF	37.000	7.298	0.029
6	NRTA	36.000	7.101	0.028
35	CBRC	32.000	6.312	0.025
29	GASC	32.000	6.312	0.025
18	MOT	27.000	5.325	0.021
26	MOC	26.000	5.128	0.020
2	MIIT	24.000	4.734	0.019
20	MOST	22.000	4.339	0.017
31	PDCPC	13.000	2.564	0.010
32	ODCPC	12.000	2.367	0.009
7	NGOA	10.000	1.972	0.008
22	MARA	10.000	1.972	0.008
27	MOEE	10.000	1.972	0.008
17	SASAC	10.000	1.972	0.008
4	SAC	9.000	1.775	0.007
9	SAMR	9.000	1.775	0.007
16	PROSC	8.000	1.578	0.006
38	CCO	7.000	1.381	0.006
11	NRA	5.000	0.986	0.004
13	SPO	5.000	0.986	0.004
34	PBC	3.000	0.592	0.002
36	CSRC	3.000	0.592	0.002
14	NATCM	2.000	0.394	0.002
8	SDB	1.000	0.197	0.001
15	SC	0.000	0.000	0.000
